# A Second Letter to the Reviewers of His Work on Dropsy, &c. Containing Additional Observations on Pathology, &c.

**Published:** 1825-10

**Authors:** Wm. Stoker


THE LONDON
### Medical and Physical Journal.

N.o 4 OF VOL. LIV.] OCTOBER, 1825. [No. 320.

For many fortunate discoveries in medicine, and for the detection of numerous errors, the world i indebted to the rapid circulation of Monthly Journals; and there never existed any work, to which the Faculty, in Europe and America, were under deeper obligations, than to the Medical and Physical Journal of London, now forming a long, but an invaluable, series.—RUSH.

### ORIGINAL COMMUNICATIONS,

### SELECT OBSERVATIONS, &c.

ART. I.—*A Second Letter to the Reviewers of his Work on Dropsy, &c. containing additional Observations on Pathology, &c.* By WM. STOKER, M.D. &c. &c.—(Communicated by Dr. JAMES JOHNSON.)

I HAVE now, in continuation of the subject of my former letter, to offer a few explanatory remarks on the cases inserted in the preface of my work on Dropsy, with a view to show why I deemed them apt for the purpose of illustration, for which I introduced them.

Scarlatina, the primary disease in the first of these cases, as one of the exanthemata, has always appeared to me to be such as very palpably evinced its origin in the fluids, as well as that the degree of morbid actions which ensue are proportioned to the alterations found to take place in the healthy condition of the blood, and its derivative fluids; and, in a former part of the same introduction, I gave a reason for the opinion that infectious and contagious diseases, attended like the exanthemata by a peculiar train of symptoms, have their origin in the fluids, in as much as the nature of the virus,—(*e. g.* in small-pox or measles,)—and not its mode of ingress into the living system, influences the result.

The colour of the surface in scarlatina on those parts where the skin is most diaphanous, and the corresponding colour of the blood, in general proportioned to the intensity of the symptoms, through the course of the disease; and the sequelæ being prognosticated chiefly from the appearances of secretions derived from the circulating mass, particularly by the black urine that precedes hydropic diathesis; are circumstances which appear to me to mark how much the state of the blood is connected with the symptoms which designate the disease of *scarlatina*.

That no organic derangement had taken place during that patient's first indisposition, I thought might be inferred from the total absence of any which could be discovered, during the long interval that occurred between it and the succeeding apoplectic attack; however, a certain degree of obesity might have indicated diseased function of the liver.

The manner* in which the patient was attacked with apoplexy,—the general stagnation of the blood which took place,—the paralysis,—and the unexpected and sudden relief of these most urgent symptoms, all seemed to me to arise from the morbid condition of the blood, connected with the disordered function of the liver; because, to the operation of the blister applied over that organ, I attributed the restoration of circulation, the flow of blood (which took place from the openings previously made), the throwing out of the latent eruption of measles, and the succeeding relief. The buffy surface on the blood drawn, too, was such as I have generally found connected with diseased function of the liver; the development of which hepatic disease (as I learned) became more obvious before those fatal dropsical effusions, which afterwards took place in the country. Indeed, was I asked to account for any of the circumstances of this case, or to answer the question of the reviewer, "how to explain the paralysis on one side?" I should think it could be done only by reference to the condition of the blood, and its derivative fluids.

In illustration of the successful application of similar principles, I beg leave to transcribe here, from my "Report of the Fever Hospital and House of Recovery, Cork-street," (for 1823,) some remarkable cases of dropsical effusion after scarlatina, which occurred to me in private practice, and were introduced into the Report to illustrate the extraordinary tendency to dropsical effusions which designated the course of the epidemic that I was then tracing; a tendency I attributed to the remarkable alterations in the condition of the blood, which I had found to accompany that epidemic from its rise, as well as that such alterations were often found materially connected, alternately, both in the relation of cause and effect, with deranged function in the hepatic system; an opinion which has been further confirmed by my subsequent observations on the course of febrile diseases, during the last two years.

About the middle of November, 1823, as I afterwards learned from Mr. Honnor, of Clare-street, one of four young gentlemen, nearly of the same age, who were cousins, and who resided together in the same lodgings in Winkworth-place, was attacked with scarlet fever, which was subsequently communicated to the three others in succession. A high degree of pyrexia, some sore throat, and vivid scarlet efflorescence,

* See *Pathological Observations*, Part I. on Dropsy, &c. 17th and succeeding pages.

were also stated to me to have accompanied the progress of disease in each; and that, under the ordinary cooling plan of treatment, the symptoms went on favourably, and convalescence succeeded at the usual periods.

On the 26th of November, however, my assistance was called for, in consequence of alarming symptoms, which came on in one of these young gentlemen, apparently connected with a dropsical tendency that had succeeded the original disease. This boy was about twelve years of age. I found him leucophlegmatic and anasarcous in every visible part; his lips were livid, and ascites to a great extent had taken place; the skin hot, dry, and rough; the liver, from its enlargement, and extending below the hypochondre, could be distinctly felt, and was sore on the slightest pressure; breathing was laborious, and expectoration difficult; rest was disturbed, unless when the head and shoulders were considerably elevated; thirst urgent, and appetite even greedy; tongue white, but moist; urine scanty, but dark coloured, the sediment like that of infused coffee. Pulse never regular; its beats at intervals slow, but very unequal, being, when I first counted them, no more than twenty-six in the minute, and afterwards thirty-six in another minute, which was the maximum of frequency during that visit. Palpitations of the heart were violent for the previous twenty-four hours, and its pulsations could be distinctly felt, and even heard, not only over the proper region of that organ, but also at the epigastrium.

Prescribed—a tepid bath for ten minutes, assisted by rubbing generally over the surface of the body and extremities with a flesh-brush; a blister to the region of the heart; a liniment of mercurial ointment, camphor, and hog's-lard, to be rubbed over the right hypochondrium twice a-day; pills, composed of blue-pill and James's powders, every sixth hour; and a powder, of eight grains of Dover's powder, at bed-time. Whey and barley-water for drink, to which may be added a dessert-spoonful of Madeira wine, when the sense of debility, of which he complained, became urgent. The temperature of the bedroom to be kept as nearly as possible at sixty degrees.

On the 27th, my second visit, there was manifest amendment in every symptom: still, however, the general dropsy, the symptoms of partial effusion into the pericardium, and those of diseased liver, demanded active remedy.

For these symptoms, mercurial friction, blue-pill combined with digitalis, assisted by small doses of compound powder of jalap, were employed.

Under the use of these remedies and a regulated diet, in twelve days every appearance of dropsy was removed, and every function was subsequently restored to its due condition: neither has there been since any interruption to the full enjoyment of this boy's health.

On the 20th of December, when paying my daily visit to another of the family, who was the last that was attacked with scarlatina, my attention was directed to the patient who is the subject of the following case, and in whom the sequelæ of the primary disease were no less alarming than in the case of his cousin, just related. It was then stated to me that this boy, twelve years of age, was the first that sickened in scarlatina, and who, being some days convalescent, had returned to school; that, on the 17th instant, however, dropsical swellings, particularly on the face and hands, appeared; but, not being attended by loss of strength, of appetite, or of sleep, these swellings did not seem to deserve any particular care; that, in the course of the last night (the 19th instant), on getting out of bed, under the influence of aperient medicine given to him the evening before, he found his vision imperfect, objects appearing double, and that he was incapable of seeing his way to bed. His urine, passed in the course of the preceding twenty-four hours, was also described to be dark, and with the same coffee-like sediment as in his cousins just detailed.

On examining, I found that anasarca had supervened to a great extent in every visible part; the colour of the skin was leucophlegmatic; the pupils of his eyes were widely dilated; the pulse was very slow, but very irregular, not exceeding fifty beats in the minute; palpitation of the heart violent; his spirits, however, were not even now depressed, and his appetite remained keen, as I learned it had been for many days before. So little did he feel himself indisposed, that he expressed much reluctance against submitting to the means I was just then directing his attendant to adopt for his cure. Slight strabismus was perceptible, and, on inquiry, I found he saw all objects double.

Prescribed—a low diet; the head to be shaved, washed with camphorated spirit of wine, and covered with flannel; a blister to the nape of the neck, and twelve leeches to the temples; pills of mercury and James's powders every sixth hour.

20th December, evening, (second visit.)—Having been summoned at nine o'clock P.M. to attend this patient, in consequence of an alarming change, I found him in convulsions. The convulsions, however, chiefly confined to the right side, were very violent and constant; and the opposite side was completely paralytic.

The face was frightfully distorted, but most so on the right side; the spasms producing constant nictitation of the eye on that side, whilst that on the other was closed. The pupils in both were widely dilated; the jaws were firmly locked by the spasm, and the mouth foamed. The pallid look of the face had entirely, and the anasarca partially, disappeared since morning. The pulse was rapid, irregular, and indistinct; breathing quick and laborious; the abdominal muscles were rigid. As he would not allow the leeches directed in the morning to be previously applied, the apothecary, who arrived some time before me, was now putting them to his temples, and using the wash to the head, which had been shaved.

Prescribed—an additional number of leeches, altogether amounting to thirty-six, two of which were to be applied inside the nostrils; warm applications to the feet and epigastrium, which were cold to the touch; a blister to the spine, and another on the right hypochondre; a liniment of mercurial ointment, oil of turpentine, and tincture of opium, to be frequently rubbed on the abdomen; enemata of turpentine, castor-oil, and saline solution, to be given every hour, till some discharge from the bowels be produced; and an ammoniated mixture, with ether and tincture of castor, to be given occasionally, as soon as the mouth can be opened, and the power of swallowing be restored.

Having remained some time to witness the effects of these remedies, I was further encouraged to direct the opening of the temporal artery; which was accordingly done, and six ounces of blood were thus taken.

At midnight, the spasms were somewhat mitigated on the right side, and some motion was observable in the limbs of the left side. The face was also less rigid, and the pulse more distinct; but, on the whole, the ground for hope was still very limited indeed.

Early on the succeeding morning, I was gratified by a message from a gentleman of Mr. Honner's establishment, who sat up with the little patient all night, informing me that the spasms and paralysis had almost entirely ceased; that the bowels had been well opened by the enemata, and a copious discharge of urine had followed; that the trismus had also subsided, and the patient had drank freely of diluents, and took his medicine well.

December 21.—On my visit at ten o'clock A.M. I was happy to find this good report fully confirmed. The patient had been sleeping on his side quietly for some time before my arrival; and, on his awaking, the only remnant of the symptoms detailed in the last report was a considerable degree of pyrexia, some head-ache and thirst. Several black stools had been passed in the night, and much urine, which was turbid, and deposited a considerable quantity of pink-coloured sediment. Blisters have risen well.

Prescribed—an aperient saline mixture, and powders, composed of James's powder and compound powder of ipecacuanha, every sixth hour; saline effervescing draughts occasionally; diluent drink.

22d December.—Fever continues high; bowels free, urine copious.

Prescribed—repeat the saline draughts; twelve drops of the tincture of digitalis, with five grains of blue-pill, three times a-day.

On the 29th, health was so fully restored as to enable the patient to join his cousins, who had gone some days before to their respective father's residences in the country, many miles from Dublin; and I have since heard that none of them have had any relapse towards their former indisposition;"* and they have continued so up to the present date, 20th July, 1825.

I deem it best to submit the foregoing quotation, without further comment, to the reader's judgment, to decide how far the observations which I have made on the first case are applicable to it. The cases it details, as already stated, are intended as examples of the hydropic diathesis, which has been so frequently excited of late years by febrile distempers, and which is to be relieved, in my opinion, by such remedies directed, as in these cases, to the condition of the animal fluids; but in the prescribing of which, however, great attention is necessary to

---

* See *Medical Report of the Fever Hospital and House of Recovery, Cork-street, for the year* 1823, page 83.

see that the typhoid fever, which often precedes such hydropic effusion, has ceased; because (as in a subsequent part of this paper will be more fully explained) evacuation, especially venesection, will be found less effectual, in proportion to the degree of that adynamic fever which may remain.

With respect to the second case quoted by the reviewers from the preface to my work, I considered it also fit to illustrate my subject, from its origin in disordered chyle, proceeding from overloaded digestion, to which succeeded disturbance in the functions of sanguification and of circulation ; all of which disturbance in the animal economy became remarkably increased on the ceasing of the vicarious discharge, which, whether a natural effort or a morbid symptom, had been for some time become habitual to this patient's constitution.

The great increase in the urgency of the symptoms, which took place on the suppression of that vicarious discharge, and all its fatal consequences, can be best understood, I think, by a reference not only to the increased plethora which succeeded, but also to the well-known effects which have been ascertained by experiments to arise from the introduction of any extraneous fluid into the sanguiferous system. Water or air, for instance, introduced into that of the horse, soon impedes the course of life. Even slight alterations in the form of the red particles, whether from some imperfection in the organs intended for their preparation or any other cause, might, I conceive, be productive of various forms of inflammatory fever ; since it has been also asserted, that if the blood of animals whose red particles are naturally spherical, be transfused into the vessels of those the red particles of whose blood are naturally oval, or *vice versâ*, that greater functional derangement, or death, succeeds more rapidly and certainly, than happens in attempts to transfuse blood with like particles in both subjects of the experiment.

In concluding those few remarks which I promised to make on the two cases just adverted to, I may be allowed to add (to the possible consequences from alterations in any part of the circulating mass,) that it has long appeared to me that the remarkable suddenness of death, which has been found frequently to happen in the various stages of hydropic effusion, and even under apparent recovery from dropsies, may be supposed to arise from the absorbed fluid passing into the sanguiferous system, and acting there as an extraneous fluid, in the same manner as in the experiments in the veterinary art just alluded to.

I know it has been, and will be, to a certain extent justly, objected to such analogies drawn from the effects of agents on the operations of life, in man and in other animals ; because of the absence of some organs, of the different configuration, magnitude, and site of others; but, above all, of the greater influence which the mere sentient principle has on organic life in the latter, than the reflecting one has in the former. But, such circumstances being kept carefully in mind, much benefit is still to be derived, I am persuaded, from comparative views of this kind, particularly as regards the animal fluids, and the effects of alterations in them of producing irregular or morbid actions in the system. The external and chemical characters of these fluids, and their mode of separation when out of the body, are very similar in all warm-blooded animals; and any alteration in their essential properties of fluidity appear to be alike attended with corresponding functional derangement. If comparative experiments, directed to this subject, were confined to the human species alone, it would be still indispensably necessary to take into account the different results from the same agents in the practical conclusions deduced from them, as for the student of nosology to consider how much the condition of the recipient is found to influence the efficacy of the dose of medicine prescribed for those labouring under disease.

As, in my former letter, I expressed my satisfaction at the notice taken of my work on Dropsy in the Medico-Chirurgical Journal, I beg leave to make a similar acknowledgment here to the editors of the Quarterly Journal of Medical Sciences, and to offer in the same feeling, as briefly as I can, some explication, that appears to me to be called for by the remarks in their Review, published in the fifth Number of their second volume, dated January last.

First, as to the division of purpura and dropsy into two distinct forms—*dynamic* and *adynamic;* "terms," says the reviewer, "which appear to be nearly equivalent to the more common ones, *active* and *passive*, at least as he applies them." I prefer the terms I made use of on that occasion to those either of *active* and *passive*, or *sthenic* and *asthenic;* not because of there being any material distinction in the etymology of those several synonimous epithets, but that I wished to employ those I had selected in a different sense to that in which the others had been more generally understood, and applied more particularly to the condition of the vascular system; whereas, by the terms *dynamic* and *adynamic*, I wish to express opposite states of the vis vitæ in all the functions of the body, vital, animal, and natural: in which sense I employed it in the distinction of the cases detailed for the illustration of my subject.

Secondly, with respect to "the extreme length of the cases," (an objection which, as in a note on the second case, at page 19, may be seen, I was willing to obviate,) I have only to reply, that I was induced to publish the course of their symptoms with a minuteness of detail which I would have deemed quite unnecessary in ordinary cases, by a conviction impressed on me, while witnessing them, that, if all the circumstances were not given in exact consecutive order, their connexion with the exciting causes, and their dependence on each other in succession as cause and effect, could not be duly perceived, so as to lead to just pathological induction, which I wish to promote, unbiassed by any preconceived theory as to the mode of agency of the causes producing such symptoms. I shall only add the great reason I have to be satisfied that, under the inveterate prejudices against those pathological principles which I maintain, in the able critiques on my work, its "practical matter"* is so fully recognised; and, if observers are thus induced to examine those facts which I have collected in support of my opinions, I do not fear of obtaining the reward of my labours, in having them esteemed, by those qualified to judge, useful in promoting medical science.

The alarming extent in which fatal cases† of typhous fever, succeeding to punctures received during dissection, in the various schools of anatomy in the United Kingdom, have been reported to have taken place within a few years past; and the illustration the accounts of these unfortunate occurrences, already published, afford to those pathological principles in general which I advocate, as well as of the views of the nature and treatment of typhous fever which I have adopted and recommended, will, I trust, justify the few remarks on that subject I propose making; and, more especially, because I hope thereby likewise to promote the deeply interesting inquiry connected with that investigation, directed to the best mode of their prevention or cure.

The very remarkable correspondence between the increased frequency of those cases, as well as the urgency of their symptoms under the varieties produced by different modifying causes,

---

* *Medico-Chirurgical Review*, vol. v. No. 17, page 166.

† I do not think it would be possible, amongst the vast multitude of cases of typhous fever with which this city has for some time so deplorably abounded, to select more exquisite examples of that disease than those published in the collection made by Dr. Duncan of those from the effects of wounds received during dissection, and in that of his own sufferings, so ably drawn up by Dr. A. T. Thomson.‡ The similarity of the mode of attack, of the symptoms, mode of treatment, and consequences, is most striking, to what I have been witnessing and reporting in our Fever Hospital, but particularly so within the last three years. In the cases I have published to illustrate the course of the epidemic, this similarity may be seen, and will be further illustrated by others noted in the progress of that epidemic. I confess, too, that I am not free from an additional feeling of interest in referring to these publications, from a persuasion that the same practical corollaries may be effectually deduced from my former and these latter statements.

‡ See *Case of Irritative Fever, &c. &c.* by A. T. THOMSON, M.D. F.L.S.—*London Medical and Physical Journal*, vol. liii. page 438.

with that of the epidemic fevers prevailing during similar and recent periods, offer *primâ facie* evidence of this identity, and of their mutual origin: and that typhoid fever has chiefly influenced the fatal result in the most malignant of these cases, is rendered further probable from what, I believe, will be admitted by most medical observers of any experience, that, during the periods just alluded to, most diseases, both acute and chronic, and even (as I have heard) important surgical operations, have been considered dangerous in proportion to the degree in which symptoms of typhoid fever supervened.

The increasing virulence and aggravation of the symptoms of typhous fever in this country, as may be seen by the latest reports on that subject, have been most remarkable within a comparatively recent period; and the frequency of the unfortunate occurrences alluded to in the following letter, with which I have been favoured by my friend, Mr. Kirby, has also increased in a remarkable degree during the same period.

"Dear Sir,—Your question is, whether wounds occurring in dissection or post-mortem examination, were formerly followed by the same unhappy consequences which are of late days so frequently known to ensue? My experience reaches as far back as the year 1798, since which period I have lived much in the dissecting-room. Yet I cannot recal to my mind a single fatal occurrence between that time and the death of the late Professor Dease: nor do I remember one so severe as to produce the least alarm. The injuries the young men received in their anatomical pursuits were disregarded, and produced no interruption of business. In a few instances an ulcer took place, slow of cure; but in general, after a little inflammation, without fever or axillary tumor, the disease subsided.

"Yours, very truly, J. KIRBY."

"*Harcourt-street.*

"Dr. Stoker, &c. &c."

That the principles which I have applied in the division of epidemic fevers, with a view to their nature and treatment, especially under the aggravated forms which they have assumed within the last two or three years, are illustrated by the unbiassed evidence of the statements already adverted to, will be more readily admitted by comparing them with the following extracts from my last Report of the Fever Hospital and House of Recovery, Cork-street, and introduced here for that purpose along with others, taken chiefly from the important article on "the Treatment of Wounds received during Dissection, by JOHN SHAW, Lecturer on Anatomy, &c. &c." inserted in the Medical and Physical Journal for May last.

"The division of fevers into *idiopathic* and *symptomatic*, and the subdivision of idiopathic into *simple* and *typhoid* fevers, is founded in the difference of their nature and origin, as follows:

"Simple fever arises from morbid actions, which do not produce any change in the structure of parts; and typhous fever from certain morbid changes primarily in the fluids, which produce morbid actions, and sometimes change of structure in the parts. The relation which the three classes of fevers bear to each other, may be illustrated by familiar examples in surgery,—the heat and redness produced by friction being taken to represent fever; and a wound, by which poison or virus was introduced, to represent typhus fever."*

"This collection" (by Dr. Duncan), says Mr. Shaw, "affords proofs in corroboration of a view which I have for some time entertained,—viz. that the effects of wounds received during dissection should be classed under two heads, forming cases which differ essentially from each other. The one seems to be of a specific nature, is attended with immediate danger, and is generally the consequence of examining a body a few hours after death; and, as far as my experience goes, most frequently follows dissections of the bodies of persons who have died of inflammation of some of the serous membranes. The other cases are more common, are less dangerous, and resemble in many respects those with which all who have attended to hospital practice must be familiar. I allude now to the consequences of wounds received in the dissecting-room, during the examination of parts which are putrid."†

The opinion, however, that the animal fluids are primarily and chiefly affected in typhoid fevers, by the generation or introduction of some poison, has not been adopted by me solely on authority,‡ or from the analogies just alluded to; but has been the result of the extensive observations on fever which I have had an opportunity of making, as well in this hospital (where, during my attendance, more than 60,000 patients were admitted,) as in private practice, and from having, in different seasons when pestilential fever prevailed, witnessed those cutaneous eruptions, internal and external hemorrhages, gangrenes, and even those glandular swellings by which such fevers, in their most exquisite forms, are characterised.§

[Dr. Stoker next proceeds to show, by quotations from Mr. Shaw's paper, that a close resemblance exists between their views and methods of treatment. But, as we have a right to suppose our readers acquainted with the opinions of the latter gentleman, as they were published in this Journal, we hope Dr. Stoker will forgive us for omitting them; particularly as a reference to Mr. Shaw's paper, and the valuable "Pathological Observations on Dropsy" of Dr. Stoker, will at once convince them of the analogy in question.—Editors.]

* *Report*, p. 22. † *London Med. and Phys. Journal*, No. 315, p. 369.

‡ See Medical Works of Dr. Meade, p. 181.

§ *Medical Report of Fever Hospitals*, by Dr. Stoker, 1823.

It would exceed the due limits of a letter of this kind, or I would willingly extend such alternate quotations from my works published on Fever, and from the statements respecting those maladies recently produced by dissection, as the analogies which it was my object to exhibit would have been thus rendered more apparent; but I must refer to the works themselves for the similarity of my practical views, whether preventive or curative, to those which have been successfully adopted in such maladies.

In my next letter, which, with your permission, shall be transmitted to you soon after I find that this has got place in your Journal, I shall detail the course of the epidemic fever in this country, illustrated by select cases and dissections.*

*York-street; Dublin.*

* We shall feel much obliged by the early transmission of this paper.—EDITORS.

